# The effects of cinnamon supplementation on adipokines and appetite-regulating hormones: A systematic review of randomized clinical trials

**DOI:** 10.22038/AJP.2022.21538

**Published:** 2023

**Authors:** Alireza Gheflati, Naseh Pahlavani, Elyas Nattagh-Eshtivani, Zahra Namkhah, Mohammad Ghazvinikor, Golnaz Ranjbar, Mostafa Shahraki Jazinaki, Abdolreza Norouzy

**Affiliations:** 1 *Department of Nutrition, Faculty of Medicine, Mashhad University of Medical Sciences, Mashhad, Iran *; 2 *Student Research Committee, Mashhad University of Medical Sciences, Mashhad, Iran*; 3 *Health Sciences Research Center, Torbat Heydariyeh University of Medical Sciences, Torbat Heydariyeh, Iran*; 4 *Metabolic Syndrome Research Center, Mashhad University of Medical Sciences, Mashhad, Iran*

**Keywords:** Adipokines, Systematic reviews as topic, Appetite regulation, Ghrelin, Leptin

## Abstract

**Objective:**

Cinnamon is extracted from the inner bark of Cinnamomum trees. Recent studies have indicated that cinnamon is a safe and cost-effective treatment for improving body weight, lipid profiles, insulin resistance, and blood pressure. This systematic review aimed to summarize the effect of cinnamon supplementation on adipokines and appetite-regulating hormones**.**

**Materials and Methods:**

This comprehensive literature search was conducted using databases such as PubMed, Scopus, ISI Web of Science, and Google Scholar up to March 2022 without any limitation. The quality of eligible studies was evaluated through the Cochrane Collaboration’s tool for assessing the risk of bias**.**

**Results:**

This systematic review included six clinical trial studies (363 participants), among which, only one study was performed on children, and two investigations were conducted on obese participants. A decreasing effect was found in the level of leptin and visfatin after cinnamon supplementation. Two out of three studies examined adiponectin levels and revealed non-significant effects of cinnamon consumption on this parameter. Two studies evaluated ghrelin levels and found an increase after cinnamon supplementation. The result of cinnamon supplementation on other biomarkers such as glucose-dependent insulinotropic polypeptide, glucagon-like peptide 1, and resistin was inconsistent.

**Conclusion:**

The result of this systematic review indicated the increasing effect of cinnamon supplementation on ghrelin levels and decreasing effect on leptin and visfatin levels. However, more clinical data are required to clarify the beneficial effects of cinnamon on adipokines levels due to the controversial findings of the studies.

## Introduction

Cinnamon is extracted from the inner bark of Cinnamomum trees, which belongs to the Lauraceae family and are widely distributed in South America, Australia, and Asia (Mousavi et al., 2020 ▶). Coumarin, cinnamic acid, eugenol, and cinnamaldehyde are the main components of cinnamon, which have anti-inflammatory, antioxidant, anti-tussive, anti-arthritic, anti-microbial, and anti-fungal properties (Shan et al., 2007 ▶; Broadhurst et al., 2000 ▶). Researchers have also shown that cinnamon is a highly effective agent in treating body weight problems, lipid profiles, insulin resistance, and blood pressure (Yazdanpanah et al., 2020 ▶; Maierean et al., 2017 ▶; Akilen et al., 2012 ▶; Hadi et al., 2020 ▶; Firouzi et al., 2021 ▶). 

Adipose tissue produces approximately 600 bioactive molecules, including adipokines, which act as endocrine and paracrine hormones (Blüher, 2014 ▶). A variety of processes including appetite and satiety, fat distribution, inflammation, blood pressure, hemostasis, and endothelial function are affected by these molecules. These adipokines primarily include adiponectin, leptin, resistin, apelin, and visfatin (Fisman and Tenenbaum, 2014 ▶; Blüher, 2014 ▶; Van de Voorde et al., 2013 ▶; Pahlavani et al., 2014 ▶). 

Leptin, visfatin, and resistin are typically pro-inflammatory, whereas adiponectin has anti-inflammatory properties (Fantuzzi, 2005 ▶; Moschen et al., 2007 ▶). The adipokine secretion pattern can reflect adipose tissue function, which is essential to determine the risk of developing metabolic and cardiovascular diseases associated with obesity (Blüher, 2014 ▶; Blüher et al., 2009 ▶). Adiponectin is an adipokine secreted by adipocytes, which plays a protective protein with antidiabetic, anti-inflammatory, and anti-atherogenic properties (Ajuwon et al., 2005 ▶; Ouchi et al., 2000 ▶). Visfatin is another adipokine associated with abdominal obesity, which raises monocyte pro-inflammatory factors (Porta et al., 2021 ▶). Ghrelin is the other adipokine, produced mainly by endocrine cells of the gastrointestinal tract, chiefly stomach cells, and involved in meal initiation (Klok et al., 2007 ▶). Leptin is a hormone secreted from adipocytes, which regulates appetite, body weight, and energy homeostasis as an essential factor in developing obesity (Farr et al., 2015 ▶; Jiang et al., 2014 ▶; Gruzdeva et al., 2019 ▶). Incretin hormones (GIP (gastric inhibitory peptide) and GLP-1 (glucagon-like peptide-1)) are gut peptides which are secreted after dietary intake and stimulate insulin secretion. The most critical effects of incretin hormones and leptin are reducing appetite and food intake, which leads to long-term weight loss (Nauck and Meier, 2018 ▶; Farr et al., 2015 ▶).

Several clinical trials have been conducted to evaluate the effect of cinnamon supplementation on circulating adipokine levels in subjects with different conditions (Dehghan et al., 2020 ▶; Borzoei et al., 2018 ▶; Maleki et al., 2020 ▶). Maleki et al. (2020) ▶ found that 56 days of treatment with cinnamon (7 mg/kg BW) in overweight women enhances adiponectin and decreases leptin levels. Further, Sfar et al. (2019) ▶ indicated that supplementation with cinnamon (8 and 4 g per day) after ten weeks of treatment reduces resistin secretion and increases ghrelin in obese diabetic males. Unlike, other studies have found that cinnamon did not influence adipokine levels significantly (Hlebowicz et al., 2009 ▶; Borzoei et al., 2018 ▶). Different sample sizes, diversity of cinnamon preparation methods, study subjects with different conditions, and other factors could explain the discrepancy in results of studies on cinnamon supplementation on adipokine levels. This systematic review summarized the available clinical trials to evaluate the effect of cinnamon supplementation on adipokine changes.

## Materials and Methods

This systematic review was conducted based on the Preferred Reporting Items for Systematic Reviews and Meta-Analyses (PRISMA) guidelines (Moher et al., 2009 ▶). The protocol of this review was registered in the International Prospective Register of Systematic Reviews (PROSPERO) database (http://www.crd.york.ac.uk/PROSPERO) with the registration code CRD42022299551.

**Search strategy**

A comprehensive electronic database search was performed in PubMed, Scopus, Web of Science, and Google Scholar to identify the relevant articles up to March 2022 using the following search query: (Cinnamomum zeylanicum OR Cinnamomum OR Cinnamomum camphora OR Cinnamomum aromaticum OR cinnamaldehyde OR Cinnamomum verum OR cinnamon OR Ceylon cinnamon OR true cinnamon OR Sri Lankan cinnamon OR cinnamon cassia OR Chinese cinnamon OR cinnamon extract OR cinnamon Bark) AND (intervention OR trial OR clinical trial OR RCT OR cross-over OR parallel OR placebo OR assignment OR randomized OR randomized OR random OR randomly) NOT (mouse OR mice OR rats OR in-vitro OR in vitro)

This study was not restricted by language, publication time, or other filters. Two reviewers (A GH and M GH) independently screened the title and abstract to exclude irrelevant studies, and another investigator resolved the disagreements. Moreover, reference lists of original articles were searched manually to find relevant studies. Furthermore, the related published articles were found after the initial search using the search alert service.

**Eligibility criteria**

The inclusion criteria for papers were those that evaluated the effect of cinnamon supplementation on humans, the impact of cinnamon consumption and its products on adipokines, and appetite-regulating hormones conducted in a clinical trial with the publication type of original article. The exclusion criteria were trials assessing irrelevant markers (lack of favorite results), reporting the results of the same studies, and observational, review, letter to editor, and animal studies. 

**Data extraction**

The data were extracted by two researchers (Z N and E N-E) and checked by the third reviewer. The extracted data included the first author's name, study design, age and gender of subjects, publication date, intervention duration, study location, sample size, intervention and placebo type, and cinnamon supplementation daily dose. Any discrepancies between the two researchers were concluded by group consultation.

**Risk of bias and quality assessment**

A tool developed by the Cochrane Collaboration was used to assess the risk of bias in five eligible studies (Higgins et al., 2011 ▶). Each study was assessed by two authors (A GH and Z N) based on the sequence generation, allocation concealment, selective reporting, blinding, incomplete outcome data, and other possible sources of bias. The potential bias judgment depends on the score obtained through mentioned domains, stratified as yes (low risk of bias), no (high risk of bias), and unclear (uncertain risk of bias). The article quality was graded as weak, fair, or good if the <3, 3, and ≥4 domains were rated as low-risk, respectively (Table 2).

## Results

**Study selection**

**Figure 1:**
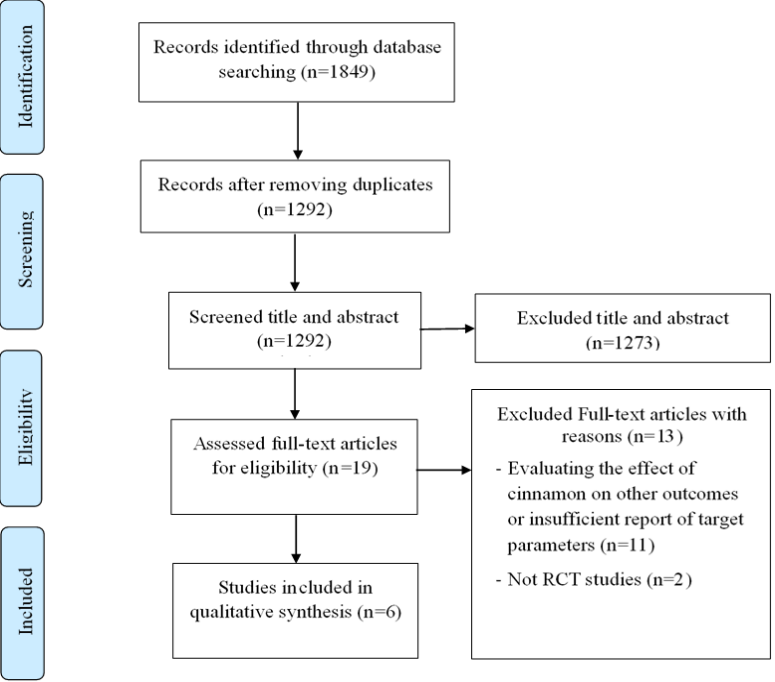
Flowchart of study selection process

Initially, 1849 articles were found through database searching with 557 duplicates. Then, 19 full-text articles were selected for further assessment after screening the titles and abstracts of 1292 remaining articles. In the following procedure, 13 articles were eliminated from the systematic review after accurately reading the remaining papers. Finally, six clinical trials were included in this systematic review, which reported the effects of cinnamon supplementation on adiponectin (three studies) (Borzoei et al., 2018 ▶; Maleki et al., 2020 ▶; Shatha Hani Mohammad, 2021 ▶), ghrelin (three studies) (Hlebowicz et al., 2009 ▶; Sfar et al., 2019 ▶; Shatha Hani Mohammad et al., 2021 ▶), resistin (two studies) (Dehghan and Abedi, 2020 ▶; Sfar et al., 2019 ▶), GIP (one study) (Hlebowicz et al., 2009 ▶), leptin (one study) (Maleki et al., 2020 ▶), and visfatin (one study) (Dehghan and Abedi, 2020 ▶)(Figure 1). 

**Study characteristics**

Table 1 presents the characteristics of eligible trials in detail. Three of the six included trials were conducted in Iran (Borzoei et al., 2018 ▶; Dehghan and Abedi, 2020 ▶; Maleki et al., 2020 ▶), one in Sweden (Hlebowicz et al., 2009 ▶), one in Tunisia (Sfar et al., 2019 ▶), and one in Iraq (Shatha Hani Mohammad, 2021 ▶). A total of 273 participants were admitted to these parallel (n=4) (Borzoei et al., 2018 ▶; Dehghan and Abedi, 2020 ▶; Maleki et al., 2020 ▶; Shatha Hani Mohammad et al., 2021 ▶), cross-over (n=1) (Hlebowicz et al., 2009 ▶), and before-after (n=1) (Sfar et al., 2019) studies. The intervention period varied from 1 to 84 days, and cinnamon dosage ranged from 1.14 to 8 g/day. There were two studies on women, one on men, two on both sexes, and one on overweight children. Eligible studies enrolled healthy participants and subjects with diabetes mellitus and polycystic ovary syndrome. The mean age of adult participants was 22.36 to 48.16 years old. Adiponectin, resistin, ghrelin, GIP, GLP-1, leptin, and visfatin levels were assessed as the primary outcome. The subjects maintained their usual diets throughout the studies.

**Table 1:** Characteristics of randomized clinical trials included in the present systematic review

**First author** **(Publication year)**	**Number and** **gender (F/M)**	**country**	**Mean age (year)**	**Study design**	**Duration** **(days)**	**Intervention** **group**	**Comparison** **group**	**Reported** **data**	**Diet type**	**Notes about** **participants**	**Result**
	84 F	Iran	Intervention (29.3) Control (30.2)	Parallel	56	500 mg capsule 3 time/day	3 placebo capsules (wheat flour)/day	Adiponectin (ng/ml)	Usual	women with PCOS BMI between 25-40 kg/m2	No significant effects on adiponectin levels
	84 M	Tunisia	48.16	Before _After	70	OAD + 8 gr of Cinnamon per day and OAD + 4 gr of Cinnamon per day	-	resistin and ghrelin	Usual	obese diabetic men	Resistin secretion decreased by 8 g dose and ghrelin increased by 4 gr dose
	9 M /6 F	Sweden	24.6	Crossover	15, 30, 45, 60,90, 120, and 150 min after the start of the meal	300 gr rice pudding mixed with 1 or 3 gr cinnamon	-	GIP, GLP-1 and ghrelin	Usual	Healthy subject (BMI = 19.3–27.5 kg.m2)	1 or 3 gr cinnamon had no significant effect on GIP, or the ghrelin response
	40 F	Iran	Intervention (22.36) Control (25.81)	Parallel	56	7 mg/kg BW cinnamon powder as capsule	Nothing	leptin and adiponectin	Usual	Healthy (BMI >25 Kg/m2 Or fat percent >35%)	Adiponectin level was increased and leptin level was decreased
	50 child	Iran	NR	Parallel	84	380 mg cinnamon capsule 3 time/day + body pump	Placebo	Resistin and visfatin	Usual	Children with overweight	Visfatin levels decreased without significant effects on resistin levels
	27 M/30 F	Iraq	NR	Parallel	84	metformin, 500mg three times daily plus crude cinnamon 1000 mg three times daily	Nothing	Ghrelin and adiponectin	usual	patients with newly diagnosed T2DM	Increase in ghrelin levels, with no significant effects on adiponectin levels

PCOS: Polycystic Ovary Syndrome/ GIP: Gastric Inhibitory Peptide/ GLP-1: Glucagon-Like Peptide-1/ BMI= Body Mass Index/ BW= Body Weight/ OAD= oral antidiabetic

**Studies on adiponectin**

Three studies examined the effect of cinnamon supplementation on adiponectin levels (Borzoei et al., 2018 ▶; Maleki et al., 2020 ▶; Shatha Hani Mohammad et al., 2021 ▶). The study duration was eight weeks in two trials (Borzoei et al., 2018 ▶; Maleki et al., 2020 ▶) and 12 weeks in one trial (Shatha Hani Mohammad et al., 2021 ▶). Borzoei et al. (2018) ▶ used 500 mg cinnamon three times a day. In contrast, Maleki et al. (2020) ▶ and Shatha Hani Mohammad et al. (2021) ▶ supplemented 7 mg/kg BW cinnamon powder and 3 g/day crude cinnamon, respectively (Maleki et al., 2020 ▶; Shatha Hani Mohammad et al., 2021 ▶). The adiponectin levels of intervention groups were not significantly different from those of control groups in the two studies. However, adiponectin level was significantly increased in the supplementation group in Maleki et al. study (2020) ▶.

**Studies on resistin**

Cinnamon supplements were examined for their effects on resistin levels in two studies (Dehghan and Abedi, 2020 ▶; Sfar et al., 2019 ▶). One of the studies was conducted on 84 obese diabetic males, while Dehghan and Abedi (2020) ▶ study was carried out on 50 overweight children. In overweight children, 380 mg of cinnamon three times a day had no significant effect on resistin levels (Dehghan and Abedi, 2020 ▶). Nevertheless, 8 g of cinnamon powder significantly decreased the resistin levels in Sfar et al. (2019) ▶.

**Studies on ghrelin**

The ghrelin response of healthy subjects after consuming rice pudding with or without 1 or 3 g cinnamon was investigated by Helbowicz et al. (Hlebowicz et al., 2009 ▶). Adding 1 or 3 g cinnamon did not significantly affect the ghrelin concentration. However, Sfar et al. (2019) ▶ found that 4 or 8 g cinnamon may significantly increase ghrelin secretion in obese diabetic men. Furthermore, Shatha Hani Mohammad et al. (2021) ▶ showed that intake of 1500 mg metformin plus 3 g cinnamon per day significantly increases in ghrelin (Shatha Hani Mohammad et al., 2021 ▶).

**Studies on other adipokines**

Helbowicz et al. (2009) ▶ studied the effect of 1 and 3 g cinnamon on plasma concentrations of incretin hormones (GIP and GLP-1) in healthy subjects with a mean BMI of 22.5 kg/m^2^. According to this study, the ingestion of 3 g cinnamon increases GLP-1 concentrations without significantly affecting GIP. Cinnamon supplementation seemed to reduce the visfatin level based on the Dehghan and Abedi (2020) ▶ study. Furthermore, leptin level was significantly changed according to Maleki et al. study (Maleki et al., 2020 ▶).

**Risk of bias and quality assessment**

The quality assessment details of studies selected in this systematic review are presented in Table 2 using Cochran Collaboration tools (Higgins et al., 2011 ▶).

As shown in this Table, four out of six studies defined the random sequencing generation method (Borzoei et al., 2018 ▶; Dehghan and Abedi, 2020 ▶; Hlebowicz et al., 2009 ▶; Shatha Hani Mohammad et al., 2021 ▶). Furthermore, only two studies described the precise method of allocation concealment (Borzoei et al., 2018 ▶; Dehghan and Abedi, 2020 ▶). In addition, participants and personnel blinding was only described in two studies (Borzoei et al., 2018 ▶; Dehghan and Abedi, 2020 ▶), and the blinding of outcome assessment was unclear for most studies except one study (Hlebowicz et al., 2009 ▶). Selective reporting or attrition bias and incomplete outcome data were not observed in all studies.

**Table 2:** Study quality and risk of bias assessment using Cochrane Collaboration's tool: ((+) means low risk of bias, (?) means unclear risk of bias, (-) means high risk of bias)

**Study**	**Random sequence generation**	**Allocation concealment**	**Blinding (participants and personnel)**	**Blinding (outcome assessment)**	**Incomplete outcome data**	**Selective reporting**	**General quality**
	+	+	+	?	+	+	Good
	?	-	-	?	+	+	Weak
	+	-	-	+	+	+	Good
	?	?	?	?	+	+	Weak
	+	+	+	-	+	+	Good
	+	?	?	?	+	+	Fair

## Discussion

According to the present review, cinnamon consumption increases ghrelin and decreases leptin and visfatin levels. However, these effects have not been conclusively demonstrated in all studies.

 Chronic conditions, such as type 2 diabetes, cardiovascular disease, and metabolic syndrome, can occur by physiological and metabolic changes in adipose tissue and energy balance. Moreover, some peptides, including leptin and adiponectin, play a pivotal role in these variations (Martins et al., 2008 ▶; Rasad et al., 2014 ▶). Adiponectin hormone regulates a wide range of biological activities in the adipose tissue, and its serum concentration decreases in chronic diseases and insulin resistance (Kelly et al., 2007 ▶). Adiponectin, produced by pancreatic beta cells, affects the liver and skeletal muscle through its R1 and R2 receptors. In addition, weight loss, calorie restriction, and increased physical activity enhance serum adiponectin levels (Haghighi et al., 2012 ▶). According to the present study, cinnamon supplementation had no significant effect on plasma adiponectin levels compared to control groups in two studies. This supplement increased adiponectin levels in the intervention group compared to baseline values only in one study (Maleki et al., 2020 ▶). Different parts of the cinnamon plant have different ratios of hydrocarbons and phenolic constituents with various active ingredients, which is one of the reasons for its various effects in various studies (Ranasinghe et al., 2013 ▶). Adiponectin secreted from adipose tissue is an influential factor in insulin resistance, which is considered a marker of obesity and diabetes and its level is reduced in these diseases (Haghighi et al., 2012 ▶). An essential property of cinnamon is that it mimics insulin, and cinnamon extract has been shown to phosphorylate insulin receptors (insulin-receptor-kinases) and inhibit their dephosphorylation and ultimately activate these receptors (Khan et al., 1990 ▶; Sangal, 2011 ▶). There are several possible mechanisms by which cinnamon affects blood sugar, including glucose uptake stimulation, insulin release, insulin receptor sensitivity, inhibition of gluconeogenesis, and decreased intestinal glucose absorption (Ranasinghe et al., 2013 ▶). In addition, cinnamon may reduce fat peroxidation by inhibiting the 5-lipoxygenase enzyme, partly because oxidative stress and inflammation contribute to diabetes (Lee et al., 2003 ▶; Domingueti et al., 2016 ▶). Previous studies have shown that increased adiponectin levels are related to improved insulin sensitivity and secretion. Cinnamon has been shown to increase glucose uptake, while adiponectin secretion was reduced in adipocytes 3T3-L1 in one study (Roffey et al., 2006 ▶). Even though cinnamon does not seem to have any significant effect on adiponectin levels, more detailed studies should be conducted with various doses of cinnamon and different durations to prove this hypothesis.

Insulin resistance appears to be caused by some adipokines, the most prominent of which is resistin which reverses the metabolic function of insulin (Blaschke et al., 2006 ▶; Reilly et al., 2005 ▶). The results showed that cinnamon could reduce resistin serum levels in obese diabetic adults, but its effect was not significant on serum resistin levels in children. In an animal study, cinnamon supplementation reduced resistin levels and decreased insulin resistance in rats (Mohamed et al., 2012 ▶). Decreased lipid profiles, such as lower cholesterol levels, seem to decrease changes in serum resistin levels (Kushiyama et al., 2005 ▶). 

Previous studies have shown that cinnamon reduces lipid profile, and inhibiting HMG-CoA reductase may be one of the most important mechanisms for lowering serum lipids (Rahman et al., 2013 ▶; Lee et al., 2003 ▶). Furthermore, cinnamon supplementation reduces leptin levels and resistance (Shalaby and Saifan, 2014 ▶; Lopes et al., 2015 ▶). Serum levels of leptin, a hormone secreted by adipose tissue, can control appetite, food intake, and energy expenditure. Cinnamon enhances leptin levels, improving metabolism, reducing obesity, and decreasing appetite (Friedman, 2011 ▶). As a result of cinnamon consumption, critical enzymes and transcription factors involved in fat metabolism are regulated, thereby reducing lipogenic processes (Lopes et al., 2015 ▶; Shalaby and Saifan, 2014 ▶). 

Visfatin is a protein secreted from visceral adipose tissue, and its levels increase in obesity and insulin resistance (Fukuhara et al., 2005 ▶). In two studies, visfatin and leptin levels decreased after administrating cinnamon supplements (Dehghan and Abedi, 2020 ▶; Maleki et al., 2020 ▶). It seems that visfatin can play a dual role in metabolism to increase the differentiation and accumulation of fat cells in visceral adipose tissue and raise insulin sensitivity in peripheral tissues (Sethi and Vidal-Puig, 2005 ▶). Therefore, more studies are required to determine the exact effects of cinnamon on visfatin levels. 

Cinnamon regulates leptin and visfatin levels by affecting cyclic adenosine monophosphate (cAMP) production and by increasing glucose transporter type 4 (GLUT-4) function, which enhances glucose absorption. Therefore, glucose can stimulate leptin secretion from adipocytes as an intracellular signal (Bahram and Mogharnasi, 2015 ▶; Souri et al., 2011 ▶).

Sfar et al. (2019) ▶ showed that consumption of 4 to 8 g of cinnamon increases ghrelin levels. However, Hlebowicz et al. (2009) ▶ by using 1-3 g/day of cinnamon found no significant decreasing effect on ghrelin levels. Another study showed that cinnamaldehyde derived from cinnamon could reduce ghrelin secretion as a transient receptor potential ankyrin 1 (TRPA1) agonist in rat gastric epithelial cells (Hafizur et al., 2015 ▶). The effects of cinnamon on ghrelin levels are contradictory, depending on the dose, intervention duration, and health status of the subjects. Ghrelin is a peptide hormone secreted from the endogenous part of the pancreas, stimulates the feeling of hunger, and acts as a leptin antagonist (Scerif et al., 2011 ▶). This small number of previous studies does not suffice to shed light on cinnamon's effects on appetite or ghrelin levels, and more studies are needed. As a result of differences in study design, health conditions, age (adults or children), and outcomes, it is difficult to determine the effects of cinnamon on appetite-related adipokines in our study.

This systematic review analyzed clinical trial studies on cinnamon and any forms of cinnamon effects on adipokine levels and appetite-regulating hormones. Although some studies have shown the increasing effects of cinnamon supplementation on ghrelin levels and its decreasing effects on leptin and visfatin parameters, these findings do not seem to indicate a precise effect on increasing or decreasing appetite or related adipokines. Detailed studies with larger sample size and long-term duration are required to evaluate these effects.

## Conflicts of interest

The authors have declared that there is no conflict of interest.
